# Novel Aspects of Nonfasting Lipemia in relation to Vascular Biology

**DOI:** 10.1155/2012/419015

**Published:** 2012-04-02

**Authors:** M. Castro Cabezas, Kathleen M. Botham, John C. L. Mamo, Spencer D. Proctor

**Affiliations:** ^1^Department of Internal Medicine, Center for Diabetes and Vascular Medicine, STZ Center of Expertise, St. Franciscus Gasthuis P.O. Box 10900, Rotterdam, BA 3004, The Netherlands; ^2^Department of Veterinary Basic Sciences, The Royal Veterinary College, Royal College Street, London NW1 0TU, UK; ^3^Centre for Metabolic Fitness, Curtin Health Innovation Research Institute, Faculty of Health Sciences, Curtin University, P.O. Box U1987, Perth, WA 6845, Australia; ^4^Department of Agriculture, Food and Nutritional science, University of Alberta, Edmonton, AB, Canada T6G 2P5

Several cardiovascular risk factors have been described during the last decades. Postprandial hyperlipidemia has been recognized as an important factor contributing to atherogenesis; however chylomicron remnants have not been used widely in risk equations. One of the probable causes for this lack of consideration in clinical practice may be that the metabolism of chylomicrons and their remnants has been rather difficult to study and, more so, to modulate in vivo. The current issue of the International Journal of Vascular Medicine provides novel insight into the metabolic changes of chylomicrons and their remnants in relation to vascular damage. The paper by G. H. Tomkin and D. Owens “*The chylomicron: relationship to atherosclerosis*” provides an excellent overview of the molecular mechanisms involved in chylomicron synthesis and metabolism in humans. The paper by B. Klop et al. “*Understanding postprandial inflammation and its relationship to lifestyle behaviour and metabolic diseases*” describes novel aspects related to inflammation in different clinical conditions and under variable lifestyle situations. Following this line of inflammatory-dependent aspects of chylomicrons, the paper by M. Cao et al. “*Dual AAV/IL-10 plus STAT3 anti-inflammatory gene delivery lowers atherosclerosis in LDLR KO mice, but without increased benefit*” further explores aspects of cholesterol metabolism involving different molecular targets important for both hepatic and intestinal cholesterol metabolism. S. A. Lopez et al. proceed with a paper investigating the effects of oxidized chylomicron remnants on monocyte activation and the effects of the antioxidant drug probucol on this interaction “*The oxidative state of chylomicron remnants influences their modulation of human monocyte activation.*” L. D. Roche and et al. provide evidence suggesting that postprandial hypertriglyceridemia simulated by an infusion with artificial TG-rich lipoproteins (Lipofundin) induces oxidative stress and atherosclerotic lesions in an animal model prone to develop atherosclerosis “*Lipofundin-induced hyperlipidemia promotes oxidative stress and atherosclerotic lesions in New Zealand white rabbits.*”

The paper by M. Napolitano et al. “*Phospholipase A2 mediates apolipoprotein-independent uptake of chylomicron remnant-like particles by human macrophages*” includes novel work showing that macrophage lipolytic enzymes mediate the internalization of postprandial TG-rich lipoproteins and that secretory phospholipase A2 (sPLA_2_) and cytosolic PLA2 play a more important role than extracellular lipoprotein lipase-mediated TG hydrolysis. M. M. Pallebage-Gamarallage et al. describe in the following investigation “*A diet enriched in docosahexanoic acid exacerbates brain parenchymal extravasation of Apo B lipoproteins induced by chronic ingestion of saturated fats,*” how dietary interventions can lead to modulation of cerebrovascular inflammation by oxidative stress. A. Van der Wiel provides a comprehensive update on the effects of alcohol ingestion on fasting and postprandial triglyceridemia “*The effect of alcohol on postprandial and fasting triglycerides*”, which is followed by a paper by B. Klop et al. “*AT1 receptor gene polymorphisms in relation to postprandial lipemia*” showing a novel relationship between polymorphisms of genes involved in the regulation of blood pressure with postprandial lipemia. J. P. H. van Wijk and M. Castro Cabezas contributed with an update on the effects of antiretroviral therapy in HIV-infected patients on TG metabolism “*Hypertriglyceridemia, metabolic syndrome, and cardiovascular disease in HIV-infected patients: effects of antiretroviral therapy and adipose tissue distribution.*” Finally, G. Charach et al. provided a review describing the effects of bile acid secretion and intestinal absorption on plasma lipoproteins and coronary atherosclerosis.

*M. Castro Cabezas* *M. Castro Cabezas* *Kathleen M. Botham* *Kathleen M. Botham* *John C. L. Mamo* *John C. L. Mamo* *Spencer D. Proctor* *Spencer D. Proctor*

